# Men’s perspectives on the impact of female-directed cash transfers on gender relations: Findings from the HPTN 068 qualitative study

**DOI:** 10.1371/journal.pone.0207654

**Published:** 2018-11-26

**Authors:** Makhosazane Nomhle Khoza, Sinead Delany-Moretlwe, Fiona Scorgie, Jennifer Hove, Amanda Selin, John Imrie, Rhian Twine, Kathleen Kahn, Audrey Pettifor, Catherine MacPhail

**Affiliations:** 1 Wits RHI, Faculty of Health Sciences, University of the Witwatersrand, Johannesburg, South Africa; 2 MRC/Wits Rural Public Health and Health Transitions Research Unit (Agincourt), School of Public Health, Faculty of Health Sciences, University of the Witwatersrand, Johannesburg, South Africa; 3 Department of Epidemiology, University of North Carolina, Gillings School of Global Public Health, Chapel Hill, North Carolina, United States of America; 4 Carolina Population Center, University of North Carolina at Chapel Hill, Chapel Hill, North Carolina, United States of America; 5 University of Wollongong, School of Health and Society, Wollongong New South Wales, Australia; Swiss TPH, SWITZERLAND

## Abstract

**Background:**

HIV is an inherently gendered disease in eastern and southern Africa, not only because more women than men are infected, but also because socially constructed gender norms work to increase women’s HIV-infection risk. The provision of cash transfers to young women alone in such a context adds another dimension to already existing complex social relations where patriarchal values are entrenched, gender inequality is the norm, and violence against women and girls is pervasive. It raises concerns about complicating young women’s relationships with their male partners or possibly even setting them up for more violence. In our attempt to understand how cash transfers influence social relations in the context of a trial among young women in South Africa, we used qualitative data collected during the trial to explore men’s perceptions of the impact of cash transfers on male-female relationships, both intimate and platonic, peer relationships.

**Method:**

Between April 2012 and August 2015, we conducted focus group discussions (n = 12) and interviews (n = 20) with the male peers and intimate partners of young women aged 13–20 years, who were participating in a phase III randomised controlled trial of CTs for HIV prevention in Mpumalanga, South Africa. A thematic content analysis approach was used to analyse the data. The codebook was developed on the basis of the topic guides, with additional codes added inductively as they emerged from the data.

**Results:**

Intimate partners were older (range 20–32 years) and more likely to be working than the male peers. Both intimate partners and male peers were supportive of the CT trial targeting young women; younger peers however expressed some concerns that the money might diminish their power and status in relationships. HIV testing requirements associated with the trial appeared to have improved communication about sex and HIV in intimate relationships, with some women even encouraging their partners to go for an HIV test.

**Conclusion:**

CTs provide AGYW with a measure of autonomy and power to contribute in their gendered relationships, albeit in limited ways. However, there is potential for CTs to have a negative impact on male-female relationships if the cash received by AGYW is equal to or greater than the income earned by their male counterparts or sexual partners.

## Introduction

High incidence of HIV among adolescent girls and young women (AGYW) in eastern and southern Africa remains a critical public health issue, with AGYW representing two out of three new infections in 2016 [1]. HIV incidence in this region is driven in part by social and economic circumstances [2] and gender inequality, including intimate partner violence [3]. Recognition that structural factors influence the risk of HIV infection and act as barriers to accessing HIV services has stimulated interest in the potential role of cash transfers (CTs) in HIV prevention and treatment [4–7]. A review of 16 CT interventions that aimed to reduce risky sexual behaviour with a view to supporting HIV prevention in nine countries showed promising results, especially when CT were targeted at young women [5]. Of three randomised controlled trials with HIV endpoints reviewed, one in Malawi (n = 1289) found that unconditional and conditional CTs reduced pregnancy and early marriage among AGYW [8]. Another trial (HPTN 068) in South Africa found that AGYW receiving CTs with school attendance as a condition were less likely to report intimate partner violence (IPV) and risky sexual behaviour, compared to their counterparts in the control arm; although, the CT did not reduce HIV incidence [9]. Similarly, another trial also in South Africa (CAPRISA 007) found that cash incentives conditional on 80% participation in a life skills program, taking an HIV test, passing academic tests, or submitting a report on their community project had no effect on HIV incidence, but did reduce HSV-2 incidence [10].

While the pathways through which CTs influence risky sexual behaviour, IPV, and ultimately HIV remain unclear, several hypotheses have emerged. One assumption, grounded in behavioural economics theory, is that CTs could reduce risky sexual behaviour through improving individuals’ socio-economic status, their access to better-quality food and education [11], facilitating access to healthier behavioural choices [12], and thereby increasing individuals’ self-efficacy and societal power and standing [13]. Evidence has also shown that CT programmes can reduce violence by alleviating day-to-day stress and conflict among couples, enhancing the psychosocial well-being of household members and the confidence and decision-making abilities of the women in those households [14]. A sub-study conducted recently among AGYW participating in HPTN 068, showed that conditional cash transfers (CCT) delayed coital debut and reducing the recipients’ number of sexual partners thereby giving them greater agency over partner choice [15].

Understanding the mechanisms by which CTs influence HIV-related outcomes is essential for refinement of HIV prevention policies [16]. One way to understand these pathways is through understanding the way in which CTs change social relations, particularly male-female relationships [6]. HIV is an inherently gendered disease in eastern and southern Africa, not only because more women than men are infected, but also because socially constructed gender norms work to increase women’s HIV-infection risk [17–19]. The provision of CTs to young women alone in such a context adds another dimension to existing complex social relations where patriarchal values are entrenched, gender inequality is the norm, and violence against women and girls is pervasive [20]. It raises concerns about complicating young women’s relationships with their male partners or possibly even setting them up for more violence.

In our attempt to understand how CTs influence social relations in the context of a trial among young women in South Africa, we used qualitative data collected during the trial to explore men’s perceptions of the impact of CTs on male-female relationships, both intimate and platonic, peer relationships.

## Methods

### Study design

We collected qualitative data as part of an ancillary study nested within the HIV Prevention Trials Network (HPTN 068) or Swa Koteka trial, which was conducted in rural Agincourt, South Africa. HPTN 068 was a phase III randomised controlled trial assessing the effect of CCT on HIV incidence among young South African women (n = 2448) aged 13–20 years. The trial provided monthly CTs to young women (R100 ≈ USD 10) and their parents/guardians (R200 ≈ USD 20), conditional on 80% school attendance. Young women were eligible to receive the cash each month in which they met the attendance criteria as long as they were eligible to attend school and up to a maximum of 3 years. The funds were deposited directly into bank accounts for the young woman and parent or guardian separately. At baseline, the participants completed an Audio Computer-Assisted Self-Interview (ACASI), before HIV test counselling, HIV and herpes simplex virus (HSV)-2 testing, and after HIV post-test counselling. Participants were seen annually at 12, 24, and 36 months until the study completion date or their planned high-school completion date, whichever came first. Each study visit included the ACASI, HIV and HSV-2 testing (if negative at the previous visit), and HIV pre-test and post-test counselling [9]. The young women in the control arm of the trial underwent similar procedures except receipt of CCTs. The study was approved by the Human Research Ethics Committee of the University of the Witwatersrand, the Institutional Review Boards of the University of North Carolina and the Provincial Department of Basic Education in Mpumalanga Province.

### Study setting

Qualitative data were collected between September 2012 and September 2015 in rural Agincourt, South Africa. Agincourt is a sub-district of Bushbuckridge, Mpumalanga province, situated near South Africa’s border with Mozambique. This location is characterised by poverty, unemployment, poor infrastructure and temporary migration to seek employment opportunities [21]. HIV prevalence in this area, among those 15 years and older, is estimated at 17.2% [22].

### Participant selection and data collection

We purposively selected two groups of men (partners and peers) to participate in focus group discussions (FGDs) and in-depth interviews (IDI). Written assent was obtained from men younger than 18 years, with written informed consent obtained from their parents or guardians at their homes. Men aged 18 years and older provided written informed consent. For both IDIs and FGDs, assent and consent forms were in English and Xitsonga, the dominant language spoken in Agincourt.

We recruited male peers (n = 72) of girls receiving the CT in Swa Koteka to participate in FGDs. To be eligible for participation, the peers had to be male, enrolled in the same secondary schools as the female CT recipients, in grades 8–12, and willing to provide consent/assent. We collected boys’ class registers from 22 participating secondary schools. Class records were captured in an Excel spreadsheet, and potential study participants were randomly selected. We visited schools to contact these potential participants, informed them about the study and invited them to participate in the FGDs. None of the participants approached declined participation.

Over a period of three years, a total of 12 FGDs were held with male peers, four per year. FGDs lasted approximately 60 minutes and comprised of 10–11 participants. Each year different young men participated in the group discussions. The themes covered included: 1) general perceptions of the CT, 2) the impact of CTs on the relationships between young men and women, and 3) the perceived impact of CTs on school attendance. The FGDs were conducted in schools (during weekends) within the villages where study participants resided. These discussions were facilitated by trained female fieldworkers; a note-taker was available to take notes during the discussions.

In addition to these FGDs, intimate partners (n = 20) of young women who had received CTs were invited to take part in an IDI. Young women who indicated having an intimate partner at their last follow-up survey were contacted by telephone. They were asked if they were willing to give their main partner a written invitation to participate in the IDIs. Interested partners then contacted the research team by sending a call-back message via a short message service (SMS); thereafter, the team called to schedule interviews.

A semi-structured interview guide was used for IDIs with intimate partners. The guide consisted of questions on 1) demographic information, 2) perceptions of the CT trial, and 3) the impact of the CT trial. The last set of issues focused on cash issues, power and decision-making in the relationship, communication, and decision-making about condom use and HIV testing. The IDIs were carried out by one of the authors (MNK), with the assistance of a trained female research assistant. The interviews took place at the participants’ homes or workplaces and lasted between 45 and 60 minutes.

Refreshments were served after completion of the FGDs and IDIs. All FGDs and most IDIs were conducted in Xitsonga; the remainder were conducted in English, as preferred by participants. Discussions and interviews were audio-recorded, transcribed verbatim, and translated into English, where necessary. The fieldworkers transcribed and translated the FGD and IDI data. For accuracy and quality checks, all transcripts were reviewed, and 10% were checked against the audio records. Transcripts were uploaded into Atlas-ti version 7.0 for analysis.

### Data analysis

A thematic content analysis approach was used to analyse the data [23]. The codebook was largely developed on the basis of the topic guides of the FGDs and IDI. However, some codes emerged inductively from the data. Two coders (MNK and JH) independently coded the transcripts, and 10% of the transcripts were double-coded to ensure inter-coder reliability; inconsistencies were discussed until the coders agreed on interpretation [24]. The codebook was updated as the themes emerged from the data. We extracted and analysed code reports on themes relating to the perceived impact of the CT programme on the relationships of female CT recipients with their intimate partners and male peers. Memos were used to summarise findings, and illustrative quotes were extracted from the transcripts, where relevant.

## Findings

### Participant overview

Our participants included male intimate partners (n = 20) and peers (n = 72). Intimate partners were aged 20–32 years; most of them had completed the 12th grade, and over half were employed and working outside the study villages. Most intimate partners had been in relationships with the young women for 2–3 years, and had fathered a child with them. These births had occurred after enrolment in the study as women were excluded from enrolment if pregnant at the time of recruitment, but not during follow-up. Only one participant was married to a woman receiving the CT. Nine partners reported having casual sexual partners outside of the main, or serious, relationship with women receiving the CT.

In contrast, the peers were slightly younger than the intimate partners: they were school-going boys aged 13–21 years and in grade 8–12. While our study design was based on a strict conceptual division between intimate partners and peers, in practice the lines between these two groups of men were somewhat blurred. Relationships between young women and their male peers were mostly platonic, but at times they became sexual in nature, as the peers often tried to attract or charm the girls into sexual relations by sharing small amounts of cash (whenever they had it) with them, or by buying them cell phone airtime and snacks at school.

In trying to understand men’s perspectives of CTs given AGYW, we discuss four themes solicited from the data; men’s perceptions of female-directed CTs; perceived impact of CTs on intimate relationships; relationships with school-going male peers; and men’s perceptions of the CTs program beyond receipt of CTs.

### Men’s perceptions of cash transfers given to young women

Both male peers and intimate partners considered receipt of CTs as beneficial for young women participating in a CT project. Peers perceived young women as having a greater need for cash, owing to their low-income family backgrounds, peer pressure and the need for enhanced social status. Consequently, they were said to engage in ‘bad things’, which one peer described as ‘dating older men because of money’. He continued: ‘they [girls] do not even stay at home because of money, sometimes they have sex with people who are infected with diseases because of money.’ Men were aware of the role that access to CTs might play in reducing young women’s vulnerability to risky sexual behaviours, for example, by reducing the need for multiple sexual partners, and dependence on transactional sex and dating of older men for financial benefits. In a FGD, one boy felt this impact was already evident:

‘I think they [CTs] have helped because some involve themselves in relationships with multiple boyfriends just to get money, and since they started getting money [from Swa Koteka], they have fewer boyfriends.' (FGD 02)

Participants reported that receipt of CTs enabled young women to meet their basic needs such as buying food and cosmetics and having pocket money for daily use at school. Despite these positive outcomes attributed to the CTs given to women, peers mentioned that CTs could elicit unintended consequences. Sometimes the girls had to travel to access the automated teller machines (ATMs) or post office; according to the boys this led to instances where recipients missed school to collect their money. They also mentioned that some young women used CTs to buy alcohol or to visit their boyfriends. Consequently, a few peers suggested that the programme should have provided food or school uniform to young women instead of cash.

### The impact of cash transfers on intimate relationships

In several interviews, partners described how young women receiving CTs had been empowered to make a financial contribution in their intimate relationships. This took the form of lending or sharing cash with partners, buying them gifts, and paying for transport to visit partners who were staying outside the study area.

‘I remember one time I was at home, and I had not received my salary; she gave me her card and told me to buy toiletries. When I paid her back, she refused and said no I must not pay her back. She also used it [cash] for transport sometimes. I remember in December, she took transport and came here [his workplace] and she said she used money from Wits [CTs]’ (Partner 008, 23 years, employed).

In some instances, the young women paid for their partners’ basic needs. Many partners, however, were uncertain about whether it was the cash from the CTs that was being used for these activities, or money from other sources.

‘Khensani (girlfriend) would sometimes pass by and find that there is no food; she would buy 10 kg of maize meal, chicken feet or something to eat. Things that I did not have like Colgate [toothpaste], soap, and so forth … She was able to cover some things in the house. She was not staying with me; she was just passing by, but she brought stuff, and it must have cost money, but I do not know from where’ (Partner 004, 26 years, employed).

For partners who stated that the young women shared the CTs with them, the women’s access to additional resources seemingly did not absolve the men of their gendered obligation to provide financially for their partners. These men reported that they continued to give the young women monthly pocket money or buy them gifts. One partner explained the local expectation that ‘men are providers and should not depend on women financially; rather, it should be women who rely on men.’ So while the CTs might have lessened the burden of responsibility on men to care for their female sexual partners, they did not completely erase men’s perceived masculine role as providers.

‘I felt great because instead of me giving her money… okay, I would give, but sometimes you find that I did not have money and she would say, “do not worry; this month I will do this and that with the R100; you do not need to worry yourself and all that”. But if I have… I did not take advantage and resolve that I would not give her [money]; I gave her’ (Partner 005, 31 years, employed).

Decisions about how to use the cash were made entirely by the women receiving the CT. Most partners displayed no interest in details of the conditions and the amount and expenditure of the CTs, stating that they did not want the women to think that they were ‘after their money.’ Partners believed that the money was for the women to use at their own discretion. ‘We never spoke about the money; I did not want to be involved.’ (Partner 002, 26 years, employed).

Although most men were supportive of women receiving the cash, they feared that women’s access to cash might somehow change the power dynamics of intimate relationships providing women with power and control that they otherwise would not be able to exert. Partner 004 remarked: ‘Sometimes if a wife has money, she becomes superior and you inferior.' Likewise, Partner 002 stated: ‘In other relationships, it happens… Let us say that she is the one who provides the man with money; sometimes she may cheat because she does not beg him [for money], he will beg her.’ However, intimate partners reported that in practice, the power dynamics in their relationship with cash recipients had not actually changed. It was a surprise to many men interviewed that despite their partners’ access to money, the relationship had continued to embody traditional gender roles and power dynamics:

‘If I told her [to do] something, she would not retaliate; like, if I said “do this and [do] that," she would not say “I can’t." When a woman has money, and a man does not, life becomes difficult because a man would not be able have rules or give his woman or wife instructions because she controls everything. Nkateko did not change’ (Partner 004, 26 years, employed).

While some men portrayed their female partners as different from ‘other women’ (since the money apparently had not changed them), others noted that the money was simply too little to trigger change, especially in relationships where men were employed.

‘When a woman has money she likes to control, it is normal. Maybe she did not want to control because it was just R100; she could not control [because] I used to carry more money. So maybe that didn’t give her a chance’ (Partner 006, 32 years, unemployed).

This participant hinted further that it was the size of the CT in relation to men’s income levels that made all the difference. He said

‘If she has more money and I do not, it would be a problem. She will not allow you to tell her anything if you are not providing. She will provide and want to control, and if she wants to control me, then there will be a problem.’

The balance of power, in other words, was maintained when the men continued to earn more than their female partners.

### How cash transfers impact on young women’s relationships with school-going boys

In contrast, male peers had a different outlook to intimate partners when it came to the symbolism and significance of money. For them, money represented an important source of power in both platonic and potentially sexual relationships. They debated among themselves who had more power or control—men or women—once women had started to receive money from the CT intervention. The peers identified this cash as a (new) source of power for women because it lessened young women’s dependency on men in general. Also, many of the peers were still trying to attract or charm these girls into sexual relationships and felt that their power to persuade women was reduced when women had their own access to resources.

The cash also meant that the boys had less control over young women, as this young man explained:

‘Things have changed; before the study, it was not a problem if you can go and pick a girl and take her to your house because she knows that in the morning I will give her R20 to buy bread, but nowadays they want nothing to do with us because they are getting money, they are buying for themselves’ (FGD 05).

As a result of these changes, they felt that young women were becoming equal to men, with some even stating that women were becoming more ‘powerful’.

‘Before Swa Koteka [CT trial], boys had power because most of the time—as I have mentioned that boys like to work so that they can get money—they always have money, because they work for themselves. So, when Swa Koteka came, these girls no longer depended on boys because they know that at month-end, they get a little amount’ (FGD 08).

‘Before this research, boys had more power than girls because boys always have money … because if someone asks one to make bricks … one would get money. But now we are all equal because we are all getting money’ (FGD 05).

Male peers expressed ambivalence about how the CTs had influenced their own relationships with young women. Some young men seemed to be happy with the ‘equality’ between men and women that had been induced by CTs. They considered the intervention as beneficial to young women who needed cash, thereby enabling them to be independent. However, some male peers did not appreciate the change, instead preferring a system in which they derived a sense of control and self-worth from having young women financially dependent on them:

‘I think that before, it was good because when a person depends on you, you can be proud of yourself and being able to control your girlfriend is nice…’ (FGD 08).

There were also feelings of rejection and inferiority among some peers. Participants stated that after commencement of the young women’s participation in the programme, they demonstrated a lack of trust towards young men, no longer mingling with their male peers, with some even ending their relationships with them, as illustrated in this participant’s remark: ‘They dump us because they now have their money' (FGD 05). This was echoed by FGD 01 participants who thought their relationships with young women had deteriorated because of the receipt of CTs:

‘As my brother [referring to another participant in the FGD] said, this money enables them to have long-distance relationships. Like maybe when she goes to collect her money, and then goes to Mkhuhlu and meets another boyfriend there. So, even if we do talk to them, they will reject us because we are now nothing to them’ (FGD 01).

The above extract suggests that CTs were seen to give young women a greater say in partnership selection—and possibly also access to a larger pool of partners from whom to choose.

### Beyond the cash: Views on the conditions and desired outcomes of the trial

Discussions with young men about the CT trial inevitably prompted remarks about the goals of the trial, namely, keeping girls in school and reducing HIV infections. Many participants felt that participation in the CT trial would positively shape the young women’s futures, since the CT encouraged—indeed, compelled—girls to attend school, lest they lose their payment.

‘It [CT] is good because it makes learners—those who get the money—attend school regularly because they know that if they do not go to school regularly, they will not get the money. So, it helped improve their school attendance’ (FGD 08).

While participants across FGDs acknowledged that the conditionality improved school attendance, a small number of young men argued that it could pressurise young women to attend school even when it was not ideal to do so—for example, when ill.

While HIV testing was not a requirement for CT receipt, it was part of the trial procedures and was therefore observed by participants to be a compulsory part of the study. The routine HIV counselling and testing provided during annual study visits was regarded by both partners and peers to be a key benefit of the young women’s participation in the trial. When men were asked to mention ‘good’ elements of the study, a common remark was that: ‘The good thing about Swa Koteka is that they are teaching the girls about HIV; they are also testing them for HIV’ (FGD 08).

An unexpected finding was that the young women’s participation in the study afforded their intimate partners an opportunity to test and know their HIV status, and that this turned out to be highly valued by men. Some partners reported that it is often difficult for men to go for an HIV test when they feel healthy. However, since the young women participating in Swa Koteka were aware of their own HIV status, men reported feeling pressure from their partners to also seek HIV testing and counselling to learn what *their* status was.

‘As men, we hate testing [for HIV] because we are… the majority of men do not like testing. If I had not met her, I would have last tested a long time ago; so, it [CT trial] did have an influence’ (Partner 016, 21 years, unemployed).

These sentiments were echoed in FGD 04, where a young man stated that CT programmes should target men because it is difficult for them to visit the clinic and undergo HIV tests. They reported financial constraints and fear of HIV positive results as barriers to testing.

‘It is too difficult to tell your parents that you need money to go to the clinic to do an HIV test… if you test [HIV] positive you will be afraid to tell them’ (FGD 04).

‘We are scared to go to the clinics and do the test because once you find out you are positive, you will be stressed that you are going to die’ (FGD 04).

Some partners were only in favour of the inaccurate notion of ‘proxy testing’, in which the young women’s HIV status is presumed to be a reflection of their own. A 32 year old partner (IP 006) stated that by testing his wife, we had also tested him indirectly because ‘her blood is my blood’:

‘When she tests, she tests on my behalf; it also helps me know what is happening. She and I are partners. So, it is obvious; we are not protecting ourselves; so, if she is [HIV] positive, it means that I am also positive; if she is [HIV] negative, it means that I am also negative. So, that is how I know my status, through her.’

Knowing or presuming to know each other’s HIV status translated into trust in intimate relationships and this was perceived by couples as justification for cessation of condom use. As this young man explained: ‘[We stopped using condoms] upon knowing that we had both tested; she told me that she tests for HIV and I showed her my results; so, from then on, I trusted her’ (Partner 007, 25 years, employed).

Improved communication about HIV and sex, as well as young women’s involvement in sexual decision-making, was widely attributed to their participation in the trial. Sexual partners reported that the young women had become comfortable talking about sex and HIV and had started to engage more with condom use decisions–something they had not done before. Partners felt that the young women’s involvement in the study distinguished them from other women who were not part of the study and felt that they could trust the former because they had recently tested for HIV. Peers noted that HIV knowledge and awareness of one’s HIV status made the girls cautious about engaging in risky behaviours, such as having multiple partners, engaging in unprotected sex and dating older men.

While the intervention overall was generally regarded as acceptable and supported by men, across the various FGDs, male peers raised concerns about the exclusion of boys from the CT programme. They felt that the programme favoured females over males, but that knowing one’s HIV status was important for everyone, and that men were also at risk of contracting HIV.

‘It would be good if this programme could be rolled out to boys because boys also want this money [they laughed]. We also want to be tested, and we do not know our statuses, and when I think of going for a blood test and not getting anything in return, it is hard; but, if I know that I have or will get R100, I will also run to do the test’ (FGD 01).

Similar to intimate partners, peers noted that many young men did not know their HIV status, and argued that inclusion in such interventions would also be beneficial for men. They bolstered their arguments regarding the unfair nature of the programme by reflecting on implications for the girls in the control arm, noting that their exclusion was also unfair, although overall the intervention was well received in the community.

## Discussion

In our attempt to understand how CTs influence social relations, we used FGDs and IDIs to explore men’s perceptions of CTs and their impact on male-female relationships, during a female-directed CT trial conditioned on school attendance and aimed at HIV prevention. We found that, overall, both intimate partners and male peers were supportive of the CT program targeting young women, in spite of concerns that the money might diminish their power and status in relationships with women. The younger, peer-aged men felt this concern more acutely than the actual partners of young women who were older and more likely to be working and have a degree of financial power.

Our findings illustrate that–from the perspective of intimate partners–receipt of CTs generally empowered young women to make a financial contribution in their intimate relationships. However, the girls’ ability to make such contributions did not challenge their male partners’ masculine role as providers, nor did it appear to change their financial power. These findings are similar to studies among adult women that have shown that economic empowerment through CTs did not necessarily translate to change in gender relations in their households [20, 25]. However, in our study, data from partners suggest that the gendered roles remained unchanged because the amount of the CTs received by young women was too little. They seemed to believe that if women received the same or greater amount of cash than they did (as ‘providers’ within the relationship), this could significantly change the power balance in women’s favour, which would be unacceptable to their partners.

While the status quo appeared to have been maintained in young women’s intimate relationships, there was more evidence that CTs were perceived to have altered power dynamics in their relationships with male peers. These men reported that once young women had access to their own money they had a measure of independence from them. Effectively, the CTs received by the girls allowed them to buy things that would have otherwise been provided by their male friends, such as airtime, snacks, and lunch at school. This was interpreted by male peers as leading to a state of greater equality between girls and boys, which most peers found undesirable as it meant that girls no longer needed their (minimal) resources, making them less attractive to the girls. Some peers even interpreted this as the reason why their attempts to propose love to girls had been unsuccessful. This finding echoes much of the literature on contemporary masculinity in South Africa, which has cautioned that some men may view women’s perceived gains as challenging to their domination over women, while other men may well embrace these changes [26–28]. In Dworkin et al.’s study conducted in six provinces in South Africa, men reported that women’s ability to secure their own material belongings made it difficult for men to attract women and assert control over them [29]. This sentiment may explain a portion of the male peers’ intimidation by young women accessing CTs in our study. As these young men were school-going, they had fewer opportunities to earn money than the older partners of the women had, and consequently, ZAR 100 became enough to pose a threat to their normative role of ‘male provider’.

A noteworthy finding of this study is the observation that the HIV testing requirements of the study appeared to have inadvertently improved communication about sex and HIV in intimate relationships, with some women even actively encouraging their partners to do an HIV test. In this study, it appears that the young women’s ability to engage in decisions about sex and HIV testing was accepted by partners and did not create conflict. This is important, since we know that communication is one of the strongest and most consistent predictors of lower risk behaviours [30], and inequitable gender norms and power dynamics often hamper communication in intimate relationships [31]. These findings suggest that linking CTs to HIV testing and other prevention services could be a real benefit in future programmes. HIV testing is valuable not only for identifying those who require treatment; it is also an important entry point for emerging biomedical HIV prevention programmes targeting AGYW. CTs could potentially be one way to accelerate uptake of HIV testing and counselling (HCT) in this population. The effectiveness of linking cash incentives to STI/ HIV testing is demonstrated in a systematic review of studies conducted in Africa and the United States, which showed that incentives increased the uptake of HIV/STI testing [32, 33]. As in most behaviour change interventions, however, there could be unintended consequences: in a study in Malawi, cash incentives linked to negative HIV test results actually increased risk behaviour in men [34]. Indeed, our own findings about male partners remaining content with ‘proxy HIV testing’ suggests that there is still some way to go in developing interventions to successfully challenge HIV misinformation among young men in this setting.

As noted here, as well as by some studies in sub-Saharan Africa [35], women’s receipt and control of CTs gives them autonomy to spend their own cash, but this does not necessarily change inequitable gender relations with intimate partners, since these are often based on culturally rooted gender norms. The shift that *did* occur, instead, was restricted to the power dynamics within platonic (and potentially sexual) relationships between young women and school-going younger men. There is clearly the potential for conflict, although this outcome was not observed in the HPTN 068 trial. Despite our concerns about violence within young women’s relationships with their intimate partners, no such violence was reported in the trial [9]. Pettifor et al. [9] found that CCTs led to fewer experiences of physical violence among adolescent recipients; also, aside from the minor teasing from peers–which happened equally in both arms–no major social harms were observed. While the purpose of the CT intervention in the Swa Koteka trial was not explicitly to alter gender norms, there is growing evidence that this is indeed what CTs for empowerment are able to do [36]–most likely because cash becomes the hook to engage recipients in a broader set of discussions. In our study, the mechanism for empowerment seems to have been greater autonomy for young women, which in turn facilitated conversations about HIV.

This study was not without limitations. Most partners reported that they were not interested in the cash, but rather in HIV testing and the knowledge they gained from the programme. While focusing on the male peers and intimate partners of recipients was necessary to limit the scope of this research, views from the cash beneficiaries and partners of non-recipients may have provided additional insights to understand whether it was the cash or the study outcomes such as HIV testing that influenced the reported changed behaviours. Views from control arm partners may have also shed some light on whether reported impacts were different or the same for recipients and non-recipients. Given that recruitment strategies for partners of young women receiving CTs were designed to protect young women who may not have disclosed their study participation to partners, this may have introduced some bias in the type of partners recruited to the study. It is possible that there was over-selection of partners who are more gender egalitarian and less likely to be perpetrators of IPV. Finally, FGDs and IDIs were facilitated by a female fieldworker/researcher, which may have influenced the responses men gave–particularly in group settings, where the presence of a woman may have elicited responses engineered to boost one’s reputation vis-à-vis other male peers.

## Conclusion

Overall, our study found that intimate partners and male peers were broadly supportive of CTs directed to young women. Notwithstanding some signs of dissatisfaction with male peers’ apparently diminished control over women, the Swa Koteka trial showed how gender attitudes are already entrenched at an early age–but also that there is a population of young people that is ready to embrace more equitable gender relations. CTs provide AGYW with a measure of autonomy and power to contribute in their gendered relationships, albeit in limited ways. However, there is potential for CTs to have a negative impact on male-female relationships if the cash received by AGYW is equal to or greater than the income earned by their male counterparts or sexual partners. These findings strengthen existing evidence that CTs given to women have a clear potential to positively influence the power dynamics within male-female relationships, but that when CTs are seen to threaten fundamental principles underlying gendered power (such as those relating to income-earning potential), unintended negative consequences–in the form of a male ‘backlash’–may be likely. Future CT interventions for AGYW need to develop innovative ways to pre-empt such consequences, and not allow this to detract from the overall aim of reducing AGYWs’ vulnerability to HIV.

## Abbreviations

AGYW: Adolescent Girls and Young Women
CT: Cash Transfer
HCT: HIV Counselling and Testing
HPTN: HIV Prevention Trials Network
IPV: Intimate Partner Violence

## References

[1] UNAIDS. AIDS info 2016 [cited 2017 17 June 2017]. Available from: http://aidsinfo.unaids.org/.

[2] Wojcicki JM. Socioeconomic status as a risk factor for HIV infection in women in East, Central and Southern Africa: a systematic review. Journal of biosocial science. 2005;37(1):1–36. 15688569 10.1017/s0021932004006534

[3] Gupta GR, Parkhurst JO, Ogden JA, Aggleton P, Mahal A. Structural approaches to HIV prevention. The Lancet. 2008;372(9640):764–75. 10.1016/S0140-6736(08)60887-9 18687460

[4] Klein C, Easton D, Parker R. Structural barriers and facilitators in HIV prevention: a review of international research Beyond Condoms: Springer; 2002 p. 17–46. 10.1097/00002030-200006001-00004 10981471

[5] Pettifor A, MacPhail C, Nguyen N, Rosenberg M. Can money prevent the spread of HIV? A review of cash payments for HIV prevention. AIDS and behavior. 2012;16(7):1729–38. 10.1007/s10461-012-0240-z 22760738 10.1007/s10461-012-0240-z PMC3608680

[6] Heise L, Lutz B, Ranganathan M, Watts C. Cash transfers for HIV prevention: considering their potential. Journal of the International AIDS Society. 2013;16(1). 10.7448/IAS.16.1.18973 10.7448/IAS.16.1.18615 PMC3752431 23972159

[7] Yotebieng M, Thirumurthy H, Moracco KE, Kawende B, Chalachala JL, Wenzi LK, et al Conditional cash transfers and uptake of and retention in prevention of mother-to-child HIV transmission care: a randomised controlled trial. The Lancet HIV. 2016;3(2):e85–e93. 10.1016/S2352-3018(15)00247-7 26847230 10.1016/S2352-3018(15)00247-7 PMC5485848

[8] Baird SJ, Garfein RS, McIntosh CT, Özler B. Effect of a cash transfer programme for schooling on prevalence of HIV and herpes simplex type 2 in Malawi: a cluster randomised trial. The Lancet. 2012;379(9823):1320–9. 10.1016/S0140-6736(11)61709-1 22341825

[9] Pettifor A, MacPhail C, Hughes JP, Selin A, Wang J, Gómez-Olivé FX, et al The effect of a conditional cash transfer on HIV incidence in young women in rural South Africa (HPTN 068): a phase 3, randomised controlled trial. The Lancet Global Health. 2016;4(12):e978–e88. 10.1016/S2214-109X(16)30253-4 27815148 10.1016/S2214-109X(16)30253-4 PMC5626439

[10] Abdool Karim Q, Leask K, Kharsany A, Humphries H, Ntombela F, Samsunder N, et al., editors. Impact of conditional cash incentives on HSV-2 and HIV prevention in rural South African high school students: results of the CAPRISA 007 cluster randomized controlled trial; abstract TUAC0101LB. 8th IAS Conference on HIV Pathogenesis, Treatment & Prevention; 2015.

[11] Fiszbein A, Schady N, Ferreira FH, Grosh M, Keleher N, Olinto P, et al Conditional cash transfers: reducing present and future poverty: Washington, DC: World Bank; 2009.

[12] UNDP. Cash Transfers and HIV Prevention Discussion paper. New York: United Nations Development Programme; 2014.

[13] Garcia M, Moore CG, Moore CM. The cash dividend: the rise of cash transfer programs in sub-Saharan Africa: World Bank Publications; 2012.

[14] Buller AM, Hidrobo M, Peterman A, Heise L. The way to a man’s heart is through his stomach?: a mixed methods study on causal mechanisms through which cash and in-kind food transfers decreased intimate partner violence. BMC public health. 2016;16(1):488. 27278935 10.1186/s12889-016-3129-3 PMC4898371

[15] Kilburn K, Pettifor A, Edwards J, Selin A, Delong S, Twine R, et al, editors. The effect of a conditional cash transfer for HIV prevention on the experience of partner violence for young women: evidence from a randomized experiment in South Africa HPTN 068 Journal of the International AIDS Society; 2017: Int AIDS Society Avenue De France 23, Geveva, 1202, Switzerland.

[16] Cluver LD, Orkin FM, Meinck F, Boyes ME, Sherr L. Structural drivers and social protection: mechanisms of HIV risk and HIV prevention for South African adolescents. Journal of the International AIDS Society. 2016;19(1). 10.7448/IAS.19.1.20646 PMC4834365 27086838

[17] Pettifor AE, Measham DM, Rees HV, Padian NS. Sexual power and HIV risk, South Africa. Emerging infectious diseases. 2004;10(11):1996 10.3201/eid1011.040252 15550214 10.3201/eid1011.040252 PMC3328992

[18] Dunkle KL, Jewkes RK, Brown HC, Gray GE, McIntryre JA, Harlow SD. Gender-based violence, relationship power, and risk of HIV infection in women attending antenatal clinics in South Africa. The lancet. 2004;363(9419):1415–21. 10.1016/S0140-6736(04)16098-4 15121402

[19] Shannon K, Leiter K, Phaladze N, Hlanze Z, Tsai AC, Heisler M, et al Gender inequity norms are associated with increased male-perpetrated rape and sexual risks for HIV infection in Botswana and Swaziland. PloS one. 2012;7(1):e28739 10.1371/journal.pone.0028739 22247761 10.1371/journal.pone.0028739 PMC3256140

[20] Brady C. Walking the Talk: Cash transfers and gender dynamics. 2011.

[21] Kahn K, Collinson MA, Gómez-Olivé FX, Mokoena O, Twine R, Mee P, et al Profile: Agincourt health and socio-demographic surveillance system. International journal of epidemiology. 2012;41(4):988–1001. 10.1093/ije/dys115 22933647 10.1093/ije/dys115 PMC3429877

[22] Gómez-Olivé FX, Angotti N, Houle B, Klipstein-Grobusch K, Kabudula C, Menken J, et al Prevalence of HIV among those 15 and older in rural South Africa. AIDS care. 2013;25(9):1122–8. 10.1080/09540121.2012.750710 23311396 10.1080/09540121.2012.750710 PMC3778517

[23] Pope C, Ziebland S, Mays N. Analysing qualitative data. BMJ: British Medical Journal. 2000;320(7227):114–6. 10625273 10.1136/bmj.320.7227.114 PMC1117368

[24] MacPhail C, Khoza N, Abler L, Ranganathan M. Process guidelines for establishing Intercoder Reliability in qualitative studies. Qualitative Research. 2015:1468794115577012.

[25] Molyneux M. Conditional cash transfers: a pathway to women's empowerment?: Pathyways of Women's Empowerment, Institute of Development Studies, University of Sussex; 2009.

[26] Hunter M. Masculinities and multiple-sexual-partners in KwaZulu-Natal: The Making and Unmaking of Isoka2003.

[27] Morrell R. Of boys and men: masculinity and gender in Southern African studies. Journal of Southern African Studies. 1998;24(4):605–30.

[28] Hunter M. Love in the time of AIDS: inequality, gender, and rights in South Africa: Indiana University Press; 2010.

[29] Dworkin SL, Colvin C, Hatcher A, Peacock D. Men’s perceptions of women’s rights and changing gender relations in South Africa: Lessons for working with men and boys in HIV and antiviolence programs. Gender & Society. 2012;26(1):97–120.

[30] Moyo W, Levandowski BA, MacPhail C, Rees H, Pettifor A. Consistent condom use in South African youth’s most recent sexual relationships. AIDS and behavior. 2008;12(3):431–40. 10.1007/s10461-007-9343-3 18228125 10.1007/s10461-007-9343-3

[31] Bhatia DS, Harrison AD, Kubeka M, Milford C, Kaida A, Bajunirwe F, et al The Role of Relationship Dynamics and Gender Inequalities As Barriers to HIV-Serostatus Disclosure: Qualitative Study among Women and Men Living with HIV in Durban, South Africa. Frontiers in Public Health. 2017;5:188 10.3389/fpubh.2017.00188 28824897 10.3389/fpubh.2017.00188 PMC5534462

[32] Lee R, Cui RR, Muessig KE, Thirumurthy H, Tucker JD. Incentivizing HIV/STI Testing: A Systematic Review of the Literature. AIDS and behavior. 2014;18(5):905–12. 10.1007/s10461-013-0588-8 24068389 10.1007/s10461-013-0588-8 PMC3966986

[33] Nglazi MD, van Schaik N, Kranzer K, Lawn SD, Wood R, Bekker L-G. An incentivized HIV counseling and testing program targeting hard-to-reach unemployed men in Cape Town, South Africa. Journal of acquired immune deficiency syndromes (1999). 2012;59(3):e28. 22173039 10.1097/QAI.0b013e31824445f0 PMC3801093

[34] Kohler H-P, Thornton RL. Conditional cash transfers and HIV/AIDS prevention: unconditionally promising? The World Bank Economic Review. 2011:lhr041. 10.1093/wber/lhr041 PMC3849819 24319306

[35] MacPhail C, Khoza N, Selin A, Julien A, Twine R, Wagner RG, et al Cash transfers for HIV prevention: what do young women spend it on? Mixed methods findings from HPTN 068. BMC public health. 2017;18(1):10 10.1186/s12889-017-4513-3 28697762 10.1186/s12889-017-4513-3 PMC5504547

[36] Dworkin SL, Blankenship K. Microfinance and HIV/AIDS Prevention: Assessing its Promise and Limitations. AIDS and behavior. 2009;13(3):462–9. 10.1007/s10461-009-9532-3 19294500 10.1007/s10461-009-9532-3 PMC3770268

